# Account of Diseases in an Eastern District of London, from the 20th of April to the 20th of May, 1807

**Published:** 1807-06

**Authors:** 


### *Account of Diseases in an Eastern District of London, from the 20th of April to the 20th of May,* 1807.

| | |
|---|---|
| **Acute Diseases.** | |
| Typhus - - - | 4 |
| Pleuritis - - - | 1 |
| Apoplexia - - | 1 |
| Angina Tonsillaris - | 1 |
| **Chronic Diseases.** | |
| Tussis - - - | 10 |
| Tussis cum Dyspnœa - | 8 |
| Pleurodyne - - | 3 |
| Asthma - - - | 1 |
| Hæmoptysis - - | 2 |
| Phthisis Pulmonalis - | 3 |
| Hydrothorax - - | 4 |
| Cephalalgia - - | 4 |
| Vertigo - - - | 3 |
| Menorrhagia - - | 5 |
| Amenorrhœa - - | 3 |
| Chlorosis - - | 4 |
| Fluor Albus - - | 7 |
| Dyspepsia - - | 3 |
| Gastrodynia - - | 2 |
| Colica - - - | 1 |
| Diarrhœa - - - | 4 |
| Obstipatio - - | 2 |
| Stranguria - - | 1 |
| Rheumatismus Chronicus | 12 |
| **Puerperal Diseases.** | |
| Menorrhagia Lochialis | 3 |
| Dolores post partum - | 2 |
| Mania - - - | 1 |
| Mastodynia - - | 4 |
| **Infantile Diseases.** | |
| Aphthæ - - - | 3 |
| Dentitio - - - | 2 |
| Rachitis - - - | 1 |
| Pertussis - - - | 1 |

The spring season of the year having now pretty far advanced, and a milder temperature of the air having succeeded the cold blasts from the north and north-east, those diseases diseases which depend so much upon this state of the weather have been gradually retiring. There are yet, however, some remains of cough and catarrh, and other affections of the respiratory organs: but, owing to the change of temperature which has taken place, even those patients who are far advanced in life, and to whom these complaints are particularly threatening, have a chance of surviving the season, which is in general fatal to persons in their circumstances.

A very severe, though less common affection of the respiratory system, a spasmodic asthma, has fallen under the observation of the reporter. This disease is not like some other diseases of the chest, particularly confined to the winter season, but occurs in persons subject to it, at all periods. It generally commences pretty suddenly by a sense of stricture on the chest, succeeded by a laborious respiration, which continues for several hours, with little or no intermission. During the paroxysm, the pulse is generally quick and weak. As breathing is very difficult, so speaking for any length of time is almost impracticable; even the few words which are necessary to express his feelings or wants, give pain to the patient.

When he is able to cough, and to discharge a quantity of mucus, a remission, more or less compleat, generally ensues; and if he falls asleep after this, he awakes tolerably free from his complaint.

During the paroxysm, æther and opium have sometimes succeeded in alleviating the symptoms, but an attention to diet and regimen in the absence of a fit, is of the first importance, in order to guard against a disease which is so liable to recur.

